# From kitchen to health: how culinary workshops influence eating habits, autonomy, and wellbeing in adults–A scoping review

**DOI:** 10.3389/fnut.2025.1653406

**Published:** 2025-08-29

**Authors:** Pâmela Gracielle da Fonseca, Lucas de Carvalho Siqueira, António Raposo, Thamer Alslamah, Najla A. Albaridi, Ariana Saraiva, Nathalia Sernizon Guimarães

**Affiliations:** ^1^Department of Nutrition, Nursing School, Universidade Federal de Minas Gerais, Belo Horizonte, Minas Gerais, Brazil; ^2^Department of Primary Health Care, University Center of Patos (Centro Universitário de Patos), Patos, Paraíba, Brazil; ^3^CBIOS (Research Center for Biosciences and Health Technologies), ECTS (School of Health Sciences and Technologies), Lusófona University, Lisboa, Portugal; ^4^Department of Public Health, College of Applied Medical Sciences, Qassim University, Buraydah, Saudi Arabia; ^5^Department of Health Science, College of Health and Rehabilitation, Princess Nourah bint Abdulrahman University, Riyadh, Saudi Arabia; ^6^Research in Veterinary Medicine (I-MVET), Faculty of Veterinary Medicine, Lisbon University Centre, Lusófona University, Lisboa, Portugal; ^7^Veterinary and Animal Research Centre (CECAV), Faculty of Veterinary Medicine, Lisbon University Centre, Lusófona University, Lisboa, Portugal

**Keywords:** culinary workshops, dietary intake, feeding behavior, nutrition education, public health

## Abstract

Inadequate dietary patterns have significantly contributed to the rise of chronic diseases, highlighting the need for effective interventions. Culinary workshops have emerged as a promising strategy by offering practical and interactive food and nutrition education that may enhance food choice autonomy and promote healthier eating behaviors. This scoping review was conducted according to the Joanna Briggs Institute guidelines and the PRISMA-ScR checklist, with a protocol registered in the Open Science Framework. Searches were performed in PubMed, Embase, Cochrane Library, and the Virtual Health Library, including intervention studies published between 2005 and 2025. Study selection and data extraction were independently performed by three reviewers. A total of 30 studies were included, most from the United States (67.6%) and Australia (23.5%), with in-person workshops lasting 4–12 weeks. Improvements were reported in food autonomy, self-efficacy, and culinary practices in 81% of studies. Over 90% reported increased consumption of fruits, vegetables, and whole grains, along with reductions in ultra-processed foods. 15 studies noted improvements in anthropometric parameters, while 14 reported clinical benefits. Quality of life was evaluated in seven studies, with significant improvements in three. Culinary workshops appear to be effective in promoting health, though more standardized studies in diverse populations are warranted.

## 1 Introduction

The global dietary pattern demonstrates significant disparities, with substantial segments of the population consuming food quantities either below or exceeding established nutritional recommendations (1). Globally, the intake of whole grains, fruits, and vegetables remains consistently below recommended levels, notably in North America and Asia. In contrast, excessive consumption of red and processed meats is particularly prevalent in Oceania (2). Such dietary imbalances have direct implications for public health, substantially contributing to the escalating prevalence of non-communicable chronic diseases (NCDs), including obesity, type 2 diabetes, cardiovascular diseases, and cancer (3).

In response to this context, it is imperative to enhance educational strategies aimed at promoting healthier, sustainable, and culturally relevant dietary choices. Culinary workshops represent one such strategy, providing an experiential, participatory, and sensory-rich environment conducive to developing culinary skills, enhancing dietary autonomy, and appreciating food consumption as both a social and political act. Existing research indicates that participation in culinary workshops correlates with improved dietary quality, reduced consumption of ultraprocessed foods, and increased intake of fresh foods (4, 5). Additionally, culinary workshops can foster communal interaction and knowledge exchange, thereby strengthening social bonds and encouraging more mindful and emotionally engaged dietary practices (6, 7).

In recent years, the concept of adequate and healthy eating has expanded beyond nutritional considerations, encompassing social, cultural, environmental, and economic dimensions. This comprehensive approach aligns with principles advocated by the World Health Organization and the Brazilian Dietary Guidelines, emphasizing health promotion and sustainable development. The intersection of health promotion and sustainable development is increasingly recognized in academic research, highlighting the necessity for an integrated understanding of social determinants influencing dietary behaviors and decisions (8).

Furthermore, the escalating concern regarding socio-environmental impacts of current food systems underscores the importance of interventions that advocate the preparation and consumption of fresh and minimally processed foods, rather than ultraprocessed alternatives. Ultraprocessed foods, characterized by their nutritional inferiority, are linked to environmental degradation, biodiversity loss, and the erosion of traditional dietary practices (9, 10).

Despite documented experiences in existing literature, comprehensive systematic reviews addressing the impact of culinary workshops across multiple health outcomes remain limited. Therefore, this scoping review aims to systematically map the existing scientific literature concerning the influence of culinary workshops on dietary autonomy, habitual food consumption, anthropometric measures, clinical conditions, and biochemical parameters. In this review, the term dietary autonomy is understood in a broad sense, encompassing related constructs such as culinary self-efficacy, confidence in food preparation, and the ability to make informed and independent food choices. Although few studies directly assessed autonomy through validated tools, many reported improvements in participants' capacities to plan, prepare, and select foods, which we interpret as indicative of increased autonomy in practice. By consolidating and systematically evaluating these findings, this review seeks to reinforce culinary workshops as a strategic tool for promoting public health and dietary equity.

## 2 Materials and methods

We conducted a scoping review based on guidelines from the Joanna Briggs Institute (JBI). This review adhered to the Preferred Reporting Items for Systematic Reviews and Meta-Analyses Extension for Scoping Reviews (PRIS-MA-ScR) checklist. The scoping review protocol was previously registered and published in the Open Science Framework (OSF; https://osf.io/3zxn2/).

Given the heterogeneity of the interventions, populations, and outcomes across the included studies, and the exploratory nature of our research question, a scoping review was considered more appropriate than a systematic review or meta-analysis. Furthermore, the lack of standardized outcome measures limited the feasibility of conducting a meta-analysis, particularly for BMI-related data.

### 2.1 Identification of the research question

The PCC acronym (Population, Context, and Concept) was used to structure the research question. The population (P) included nutritional intervention programs and strategies; the context (C) was culinary workshops; and the concept (C) encompassed food services, public policy actions and strategies related to health and nutrition, community projects, or sustainable public initiatives.

### 2.2 Information search

To identify eligible studies on characteristics of nutritional intervention programs that focused on using fresh and minimally processed foods for culinary preparations, we searched primary scientific research from four electronic databases (PubMed, Embase, Cochrane Library, and the Virtual Health Library platform, reporting to Lilacs) on February 13, 2025. Local studies were identified through gray literature searches, including Connect Papers, and manual searches of reference lists of selected studies.

Search strategies were developed by an expert and refined through team discussions. Databases such as Medical Subject Headings (MeSH), Emtree, and Health Sciences Descriptors (DeCS) were consulted, and the approach was tailored to each database. The finalized PubMed search strategy and its adaptations for other databases are detailed in Appendix 1. Filters validated for intervention studies (McMaster) and publication years (2005–2025) were applied.

Following the search, studies were imported into Rayyan Qatar Computing Research Institute (Rayyan^®^) a collaborative tool for article screening, and blindly reviewed by two reviewers (PGdF and LdCS). Discrepancies were resolved by a third reviewer (NSG).

### 2.3 Eligibility criteria

Studies included were intervention studies (randomized and/or non-randomized clinical trials), quasi-experimental research, experimental trials, or mixed-methods research. Eligible studies evaluated culinary workshops aimed at enhancing dietary autonomy, dietary practices (participants' habitual food consumption), and health outcomes. Exclusion criteria comprised literature reviews, pilot studies, protocols, conference abstracts, editorials without initial results, studies involving children and adolescents, and culinary workshops not explicitly designed to promote the consumption of fresh and minimally processed foods.

### 2.4 Study selection and data extraction

To ensure consistency, two reviewers (PGdF and LdCS) participated in both the initial screening of titles and abstracts and the full-text evaluation. Disagreements were resolved by a third reviewer (NSG). Data extraction was conducted by three reviewers using an Excel spreadsheet, capturing variables detailed in Tables 1–3.

**Table 1:** Study information: journal, location, population, setting, mode of delivery, frequency, and follow-up duration.

**References**	**Journal**	**Location**	**Population**	**Setting**	**Mode of delivery**	**Frequency**	**Follow-up duration**
Castagnetta et al. (11)	Annals of the New York Academy of Sciences	Palermo, Sicily, Italy	Postmenopausal women	Tourism school in Palermo	In-person	Weekly over 1 year	1 year, divided into two phases
Pierce et al. (12)	The Journal of American Medical Association (JAMA)	Various locations, California, USA	Women with early-stage invasive breast cancer	Multisite clinical study	In-person	Monthly for 12 months	12 months
Newman et al. (13)	Journal of the American Dietetic Association	La Jolla, California, USA	Women previously treated for breast cancer	University	In-person	Monthly, 12 total classes	12 months
Brown et al. (63)	Journal of Nutrition Education and Behavior	Stillwater, Oklahoma, USA	Youth and adults	Community kitchens	In-person	Weekly, eight classes over 2 months	2 months
Wrieden et al. (14)	Public Health Nutrition	Dundee, Scotland, United Kingdom	Adults living in socially deprived areas	Community	In-person	Weekly for 7 weeks	6 months
Cutler et al. (15)	Journal of Primary Health Care	Various regions, New Zealand	Overweight women	Not specified	In-person	Weekly for 6 weeks, 2 h/session	12 months, with four evaluations
Flynn et al. (16)	Journal of Hunger & Environmental Nutrition	Providence, Rhode Island, USA	Clients of community organizations	Community food distribution organizations	In-person	Weekly, ~2 h	6 weeks
Cuy Castellanos et al. (17)	Journal of Hunger & Environmental Nutrition	Scranton, Pennsylvania, USA	Low-income adults attending farmers' markets	Farmers' market	In-person	Weekly, ~4 h	15 weeks
Bennett et al. (18)	Journal of Intellectual Disabilities	Dublin, Ireland	Adults with mild to moderate intellectual disability	Community center	In-person	Six sessions of 2.5 h; five courses total	~6 weeks
Dannefer et al. (19)	Journal of Nutrition and Behavior	New York, USA	Low-income adults attending farmers' markets	Farmers' market	In-person	Weekly from July to November	5 months
Greenlee et al. (20)	Journal of the Academy of Nutrition and Dietetics	New York, USA	Hispanic breast cancer survivors	University teaching kitchen	In-person	Weekly, nine sessions	12 months (0, 3, 6, and 12 m evaluations)
Hutchinson et al. (21)	Public Health Nutrition	Leeds, United Kingdom	Adults with limited culinary skills	Teaching kitchen	In-person	Weekly for 8 weeks, 90 min/session	Post-course and 6 months follow-up
Bernardo et al. (22)	Appetite	Florianópolis, Santa Catarina, Brazil	University students	University teaching kitchen	In-person	Weekly, five culinary classes	Before, after, and 6 months
Turner-McGrievy et al. (23)	Current Developments in Nutrition	Columbia, South Carolina, USA	Overweight adults	Clinic	In-person	Weekly for 12 weeks + monthly reinforcement	12 months
Derose et al. (24)	American Journal of Health Promotion	South Los Angeles, California, USA	African American and Latino adults	Churches	In-person	Weekly, 5 weeks	5 months
Diallo et al. (25)	Public Health Nursing	Richmond, Virginia, USA	Adults and elderly	Activity center	In-person	Weekly for 8 weeks	8 weeks
Miller et al. (26)	Nutrients	Glenside, Pennsylvania, USA	Adult cancer survivors	Clinic/university	In-person	Weekly for 8 weeks	15 weeks
West et al. (27)	Nutrients	Sydney, Newcastle, Melbourne, Australia	Low SES adults	Community services	In-person	Weekly for 6 weeks, 15 h total	6 weeks
Begley et al. (28)	International Journal of Environmental Research and Public Health	Perth, Australia	Low to medium-income adults	Online sessions	Virtual	Four sessions of 2.5 h	Pre, post, and 3 months
Reicks et al. (29)	Journal of Nutrition Education and Behavior	St. Paul, Minnesota, USA	General adults	Community and health centers	In-person	Weekly, six classes	6 weeks
Asher et al. (31)	Journal of Human Nutrition and Dietetics	Callaghan, NSW, Australia	Professionals in health, education, horticulture	Online course	Virtual	Weekly, five modules	5 weeks
Rainville et al. (32)	Journal of Hunger & Environmental Nutrition	Hamtramck, Michigan, USA	Low-income residents	University health center	Virtual	Weekly, four classes	4 weeks
Ylitalo et al. (33)	Nutrients	McLennan County, Texas, USA	Low-income adults	Clinic	In-person	Four to six sessions	6 weeks
Begley et al. (30)	Journal of Nutrition Education and Behavior	Perth, Australia	General adults	Mixed (not specified)	Hybrid	Weekly, four classes	3 months follow-up
Williams et al. (34)	Journal of the Academy of Nutrition and Dietetics	Columbus, Ohio, USA	Adults with diabetes	University	In-person	Weekly for 6 weeks	6 weeks + 3-month evaluation
Kearsey et al. (35)	Journal of Human Nutrition and Dietetics	Australia (multiple locations)	Low-income adults at risk of food insecurity	Community centers	In-person	Weekly, 2.5 h for 6 weeks	Immediate and 6 months
Barr-Porter et al. (36)	International Journal of Environmental Research and Public Health	Lexington, Kentucky, USA	University students	Teaching kitchens	In-person	Four sessions, 90 min each	16 weeks
French et al. (37)	Nutrients	Berkeley, California, USA	University students	Teaching kitchen	In-person	Weekly, 14 classes	14 weeks
Domper et al. (38)	Nutrients	San Sebastian, Spain	Overweight and obese adults	Online sessions	Virtual	4 weeks, two sessions per week	4 weeks
Heredia et al. (39)	Journal of Nutrition Education and Behavior	Houston and Dallas, Texas, USA	Adults with type 2 diabetes	Videoconference	Virtual	Biweekly or weekly, five sessions	6 months

### 2.5 Synthesis of results

A narrative synthesis was conducted, summarizing study characteristics and organizing results by year of publication. Data were systematically presented in Tables 1–3. Table 1 provides an overview of the general study information, including study references, publication period, country, workshop setting, presentation mode (in-person, virtual, or hybrid), target population, frequency of workshops, and follow-up duration. Table 2 focuses on the specifics of the culinary interventions, such as the fresh foods used, culinary preparations, food consumption patterns, and dietary autonomy, with measures like self-reports and Food Frequency Questionnaires (FFQ). Table 3 summarizes the health outcomes, including changes in anthropometric measures, quality of life, and clinical indicators, alongside the tools used for these assessments, such as self-reported questionnaires and the SF-36.

**Table 2:** Study information: foods, culinary preparations, food consumption, autonomy in food choices, and main results.

**References**	**Foods**	**Culinary preparations**	**Food consumption**	**Main results**	**Autonomy in food choices**	**Main results**
Castagnetta et al. (11)	Ingredients from the mediterranean diet were used, such as whole grains, fish, vegetables, olive oil, cheeses, and dairy products, and the participants were instructed to avoid refined carbohydrates, salt, and animal fats	The culinary preparations incorporated typical mediterranean diet recipes, featuring dishes based on legumes, whole grains, vegetables, and fish; however, specific recipes were not detailed	Assessed through a food frequency questionnaire and 24-h dietary recalls	There were significant improvements in the consumption of healthy foods, such as fruits, vegetables, and fish (*p* < 0.001)	Culinary skills were analyzed, but there is no mention of the use of specific instruments for their assessment	The participants acquired culinary skills and knowledge to independently follow the mediterranean diet
Pierce et al. (12)	A wide variety of vegetables and fruits was encouraged, aiming for a high-fiber, low-fat diet, although the study did not specifically detail the ingredients	Specific preparations were not described, but the focus was on promoting a diet rich in vegetables and fruits with a low fat content	Assessed through 24-h dietary recall	An increase in vegetables, fruit, and fiber intake and a reduction in fat intake were observed	Not reported.	—
Newman et al. (13)	Vegetables, fruits, whole grains, and high-fiber foods (e.g., dark leafy greens, citrus fruits)	Quick meal recipes, healthy dishes, use of nutrition labels, preparation of potlucks, and healthy eating at parties	Measured through self-reports and 24-h dietary recalls validated with tools such as NDS-R	The intervention resulted in increased intake of vegetables, fruits, and fiber, as reported by participants. There was also a reduction in fat consumption	Assessed by adherence to the protocol, attendance at cooking classes, and diet adherence scores (WHEL adherence Score)	Higher diet adherence was associated with greater participation in cooking classes. The average adherence score increased from 249 to 419 points over 12 months
Brown et al. (63)	Fruits and vegetables	Included salads, soups, vegetable dishes, smoothies, and healthy snacks	Measured through self-reported questionnaires	The study showed a significant increase in the consumption of fruits and vegetables. The average number of servings consumed daily increased for both youth and adults	Measured through self-reported questionnaires before and after the intervention	There was an increase in participants confidence to prepare food safely and healthily
Wrieden et al. (14)	Fruits and vegetables	Preparations included pasta bake, soup, minced meat-based dishes, rice, pizza, chicken curry, and healthy cakes	Measured through food diaries and food frequency questionnaires (FFQ)	Increase in the frequency of fruit consumption, but not sustained at 6 months	Measured through questionnaires assessing confidence in preparing and using basic ingredients	Significant increase in kitchen confidence, with reports of preparing more meals from basic ingredients and new recipes
Cutler et al. (15)	Fruits, vegetables, whole grains, low-fat dairy products, and healthy fats such as olive oil	Healthy dishes, including salads, healthy desserts, snacks, and balanced meals based on vegetables and other foods recommended by a healthy diet	Measured through food frequency questionnaire (FFQ)	Increased consumption of fruits and vegetables and reduced fat intake in the intervention group	Confidence in adopting a healthy diet, measured through self-assessment questionnaires	Reported increase in confidence to adopt a healthy diet
Flynn et al. (16)	Plant-based ingredients, such as vegetables, fruits, and extra virgin olive oil	Simple but unspecified plant-based recipes	Measured through self-report, food habits questionnaire, and supermarket purchase records	Significant increase in the consumption of vegetables and fruits, with 78% of participants reporting higher vegetable intake and 44% reporting higher fruit intake after follow-up	Measured through self-report and questionnaire on changes in food purchases and consumption after the program	68% of participants reported using a food pantry at the beginning of the program, decreasing to 54% at follow-up. The food insecurity score (FIS) improved, with a significant reduction in the number of participants classified as food insecure
Cuy Castellanos et al. (17)	Fruits and vegetables	Various preparations, but not specified	Measured through a block fruit and vegetable screener, estimating the frequency of fruit and vegetable consumption	Fruit and vegetable consumption increased from a “good” to an “excellent” score based on the Screener	Measured through an adapted questionnaire assessing the ability to access and prepare foods	Participants reported an increased ability to prepare healthy foods and incorporate them into their daily diet
Bennett et al. (18)	Ingredients not specified in detail but referring to healthy foods	Preparation of healthy meals, including learning to cook simple dishes	Not specified; evaluation was mainly based on qualitative interviews	Participants mentioned they began eating more fresh foods and making healthier food choices	Measured through self-report, where participants reported their ability to cook independently	Increase in perceived autonomy in meal preparation
Dannefer et al. (19)	Fresh seasonal produce, focusing on local fruits and vegetables	Preparations consisted of simple and healthy dishes emphasizing the use of fruits and vegetables, without specific names provided	Measured through food frequency questionnaire (FFQ) and participants' self-reports on the number of fruits and vegetables consumed	There was an increase in daily fruit and vegetable consumption among participants who attended the workshops	Measured through a self-efficacy scale	Participants showed greater self-confidence in preparing and consuming fruits and vegetables after the classes
Greenlee et al. (20)	Fruits, vegetables, and lean proteins	Traditional dishes adapted with a focus on vegetables and fat reduction, using healthier cooking methods	Measured by 24-h dietary recalls	Increased intake of fruits and vegetables	Not reported	—
Hutchinson et al. (21)	Fruits, vegetables, whole grains, and lean proteins	Healthy and simple preparations, such as soups, salads, and vegetable-based dishes, with an emphasis on reducing salt, fat, and sugar in recipes	Measured through food frequency questionnaires (FFQ) and self-assessment questionnaires	There was an increase in the consumption of fruits, vegetables, and water, and a decrease in the consumption of ultra-processed foods	Confidence in preparing healthy foods assessed through self-assessment questionnaires	Participants showed an increase in their confidence to prepare healthy foods
Bernardo et al. (22)	The study did not specify the exact ingredients used during the culinary sessions	Preparations were not specified	Self-assessment questionnaires	Increased self-efficacy in using fruits and vegetables and increased accessibility and availability of these foods at home	Cooking confidence measured on a five-point scale from “not confident” to “extremely confident”. Culinary techniques knowledge assessed by eight multiple-choice questions. Attitudes toward healthy eating assessed using a five-point likert scale from “strongly disagree” to “strongly agree”	Improvements were reported in cooking confidence, culinary knowledge, and attitudes toward healthy eating
Turner-McGrievy et al. (23)	Fruits, vegetables, legumes	Low-inflammatory index recipes	24-h dietary recalls applied on weekdays and weekends, with interviews conducted by nutritionists, including visual evaluation of food portions validated by NDSR software	The study showed that participants in the intervention group, following an anti-inflammatory diet, had a significant reduction in the Dietary Inflammatory Index (DII) after 3 months, indicating temporary improvement in food choices toward healthier options	Measurement of food autonomy was carried out through self-reported questionnaires covering eating habits, preferences, and food preparation skills	Participants' food autonomy, promoted through cooking classes and nutrition education, led to healthier food choices and a reduction in DII scores
Derose et al. (24)	Fruits, vegetables, whole grains, and lean proteins	Healthy meals such as salads, soups, vegetable dishes, and healthy snack options	Measured by food frequency questionnaires (FFQ) and self-assessment questionnaires	Significant increase in fruit and vegetable consumption and improvement in diet quality among participants in the intervention group	Measurement was carried out through self-assessment questionnaires	Increased self-efficacy and confidence in preparing healthy foods and maintaining a balanced diet were observed
Miller et al. (26)	Whole grains, vegetables, fruits, beans	Salads (e.g., Quinoa salad, southwestern bean salad), smoothies (e.g., everyday green smoothie), practical meals (one-pot meals), and other vegetarian dishes	Measured using the dietary screener questionnaire (DSQ), reduced NHANES version, estimating daily intake of fruits, vegetables, whole grains, and processed meats	Significant reduction in processed meat intake (*p* < 0.05) and increased consumption of plant-based foods and grains	Indirectly measured through three scales: confidence in preparing healthy foods, skills for maintaining a plant-based diet, perceived control over cancer progression	Significant increase in confidence by week 15 (*p* < 0.05); culinary skills also significantly improved (*p* < 0.05); trend toward improved perceived control over cancer
Diallo et al. (25)	Vegetables, fruits, whole grains, and lean proteins	Preparation of recipes focused on low salt, sugar, and fat content, though specific preparations were not detailed	Measured by food frequency questionnaires (FFQ)	Significant increase in fresh vegetable consumption among participants	Measured through self-assessment questionnaires	Participants reported increased confidence in preparing healthy foods
West et al. (27)	Unprocessed ingredients (not specifically detailed)	Preparation of healthy meals not specified	Measured through self-report and 24-h dietary recall	Significant increase in vegetable consumption and significant reduction in the intake of sugary beverages and salty snacks	Measured through self-report	Participants felt greater control over their food choices
Begley et al. (28)	Ingredients not specified	Recipes prepared were not detailed	Self-reported dietary behavior questionnaires	Reported improvements in vegetable consumption	Food literacy and culinary confidence were assessed through self-report questionnaires at the beginning, end, and 3 months after the program	Improvements or maintenance in food literacy skills and improvements in culinary confidence were reported
Reicks et al. (29)	Plant-based foods, including leafy greens, whole grains, beans, and tubers	Two to three recipes per lesson, focusing on food groups from the African heritage diet pyramid	Measured through food frequency questionnaire (FFQ) and self-reported food intake	Significant increase in the frequency of fruit, vegetable, and greens consumption after participation in the program	Measured through direct and open-ended questions on the frequency of food preparation and consumption	Increased confidence and creativity: greater use of natural seasonings, reduction in salt, preparation of vegetable and Afrocentric dishes. 98% reported cultural motivation
Asher et al. (31)	Emphasized different methods of vegetable preparation, but specific ingredients were not detailed	Preparations not specifically described	Pre- and post-program questionnaires	Statistically significant increases observed in vegetable, fruit, and bread/cereal consumption after the intervention	Confidence in food skills and nutritional knowledge measured through pre- and post-program questionnaires	Significant increases and large effects observed in confidence in food skills and nutritional knowledge scores
Rainville et al. (32)	Quinoa, spinach, assorted vegetables, ingredients for spinach and feta dips, potatoes, etc.	Toasted mediterranean quinoa and spinach, chopped colorful vegetable salad, green beans and red potatoes, creamy spinach and feta dip	Measured through self-report	Increased consumption of fruits, vegetables, and whole grains, with 100% of participants adopting the habit of reading food labels	Measured through self-report	At the end of the program, participants reported increased confidence and cooking skills
Williams et al. (34)	Fresh fruits and vegetables, including leafy greens	Healthy dishes such as salads and cooked meals	Measured through self-report and 24-h dietary recall	Significant increase in vegetable consumption and significant reduction in sugary beverage and salty snack intake	Indirectly evaluated through dietary self-care scores (SDSCA, DSMQ) and perceived control via the self-efficacy scale; questions on recipe preparation and label reading	Significant increase in SDSCA diet score post-intervention (+1.49, *p* = 0.014); DSMQ diet control score also increased (+0.72, *p* = 0.013)
Ylitalo et al. (33)	Fresh produce (fruits and vegetables)	Food preparation (including shared meals)	Measured through self-report on fruit and vegetable consumption with visual aids	Increase in vegetable intake to ≥2 cups/day from 25 to 38% of participants (*p* < 0.001); fruits: increase from 19 to 38% (not statistically significant)	Measured through validated scales: behavior change strategies for healthy eating scale (BCSHES) and culinary self-efficacy (12 items assessing confidence, skills, and attitudes toward healthy meal preparation)	Significant increases observed: culinary self-efficacy (Δ = +0.6 points, *p* < 0.001); Dietary management (Δ = +0.7 points, *p* < 0.001)
Begley et al. (30)	Vegetables were used as ingredients, but not specified	Preparations were not detailed, only mentioned the use of vegetables for main meals and snacks	Measured by the food literacy behavior checklist (FLBC) and semi-structured questionnaire	Participants increased their average daily vegetable consumption by 22.6% (0.5 servings)	Food literacy assessed through the FLBC, evaluating behaviors related to planning, selecting, and preparing foods; increase in vegetable consumption measured by questions on fruit and vegetable portions consumed, recorded during program assessments	Participants reported significant improvements in two food literacy domains: planning and management (+12.4%) and preparation (+9.8%)
Barr-Porter et al. (36)	Ingredients not specifically detailed	Prepared recipes were not detailed	Healthy eating index “short healthy eating index” (sHEI)	Increase in total vegetable consumption and general improvements in diet quality	Not reported	—
French et al. (37)	Fruits, vegetables, and lean proteins	Participants prepared vegetable-based dishes, soups, salads, and quick meals	Measured by food frequency questionnaires (FFQ)	Increase in vegetable and fruit consumption among participants in the intervention group	Measured through self-assessment questionnaires	Increased food confidence observed among participants, with improvements in the ability to prepare meals and adopt healthy eating habits
Kearsey et al. (35)	Fruits, vegetables, lean proteins, and whole grains	Preparations included simple and healthy dishes such as soups, salads, vegetable meals, and whole grain-based dishes	Measured by food frequency questionnaires (FFQ) and self-assessment questionnaires	Increase in fruit, vegetable, and water consumption and decrease in ultra-processed food intake	Culinary confidence and meal planning confidence measured through self-assessment questionnaires	Increased culinary confidence among participants, with greater confidence in cooking and meal planning
Domper et al. (38)	Mediterranean diet ingredients, such as legumes, fruits, proteins, olive oil, and whole grains	Preparations included typical Mediterranean dishes such as pesto, green curry, baba ganoush, vegetable salmorejo, and fruit ceviche	Measured by food frequency questionnaires (FFQ)	Reported increase in the consumption of vegetables, legumes, and nuts among participants in the culinary intervention group (CIG)	Culinary self-confidence and attitudes toward cooking measured through self-assessment questionnaires	Observed increase in confidence to cook at home and modify recipes to make them healthier
Heredia et al. (39)	Fruits, vegetables, whole grains, lean proteins, and healthy fats	Soups, salads, vegetable-based dishes, and healthy recipes	Measured through food frequency questionnaires (FFQ) and 24-h food records	Observed increase in the consumption of fruits and vegetables among participants, particularly in whole grains and lean proteins	Confidence in cooking healthy foods, culinary skills, and diet control measured through self-assessment questionnaires	Participants reported increased confidence in preparing healthy foods and managing their diets

**Table 3:** Study information: health outcomes, anthropometric measures, quality of life, and main results.

**References**	**Health outcomes**	**Main results**	**Anthropometric measures**	**Main results**	**Quality of life**	**Main results**
Castagnetta et al. (11)	Control of cholesterol levels	A reduction in cholesterol levels among participants was observed	Body weight, waist circumference, and hip circumference were measured before and at the end of the study	A significant weight loss was observed in the group that followed the Mediterranean diet during the follow-up period	Self-report	Participants reported an increased sense of psychophysical wellbeing after the intervention
Pierce et al. (12)	Occurrence of new breast cancer events and survival of previously treated participants	No significant differences were found in breast cancer recurrence or mortality between the intervention and comparison groups. Rates of invasive cancer events were similar across groups	Not available	—	Quality of life was assessed using the “thoughts and feelings questionnaire,” focusing on psychosocial functioning, although the main focus of the study was on health outcomes related to cancer recurrence and mortality	Quality of life results were not detailed in the results section, possibly because they were not a priority outcome or did not show significant differences considered worthy of highlight by the authors
Newman et al. (13)	Blood carotenoid levels	Results validated by an increase in blood carotenoid levels	BMI and nutritional risk assessed through the elderly nutrition screening questionnaire	Mean BMI at baseline: 26.7 kg/m^2^. Weight change was not reported as a main outcome nor analyzed in detail in the results	Not available	—
Brown et al. (63)	Not available	—	Not available	—	Not available	—
Wrieden et al. (14)	Not available	—	Not available	—	Not available	—
Cutler et al. (15)	Cholesterol levels	A reduction in LDL cholesterol and total cholesterol levels was recorded	Body weight and waist circumference	No significant changes were found in body weight or waist circumference during the follow-up period	Self-report	Improvements were observed in aspects related to quality of life, such as psychological wellbeing and satisfaction with exercise practices
Flynn et al. (16)	Not available	—	Weight and Height/BMI; Waist circumference	There was a significant reduction in BMI (from 33.3 ± 8.5 to 32.9 ± 8.4, *p* = 0.05) and in waist circumference (from 96.2 ± 16.8 cm to 95.3 ± 16.2 cm, *p* = 0.05). Forty-nine percent of participants lost an average of 5.0 kg (*p* = 0.04)	Not available	—
Cuy Castellanos et al. (17)	Not available	—	Not available	—	Not available	—
Bennett et al. (18)	Not available	—	Not available	—	Indirectly measured through participant feedback on satisfaction and self-efficacy	Participants reported feeling more capable and satisfied with their culinary skills and social life
Dannefer et al. (19)	Diabetes and cholesterol control and improved digestion	Participants reported health improvements as a result of participating in the program workshops, including better control of diabetes and cholesterol, improved digestion, and weight loss	Body weight and BMI	The study did not report significant changes in anthropometric measures such as weight or BMI	Not available	—
Greenlee et al. (20)	Not available	—	Body weight, BMI, and waist circumference	A reduction in waist circumference was observed among participants in the intervention group, but no significant changes were found in body weight or BMI	Not available	—
Hutchinson et al. (21)	Not available	—	Not available	—	Self-report	Participants reported improvements in overall wellbeing due to increased culinary confidence and improved eating habits
Bernardo et al. (22)	Not available	—	Not available	—	Not available	—
Turner-McGrievy et al. (23)	Dietary inflammatory index (DII), inflammatory biomarkers (CRP, interleukin-6, and TNF-alpha), lipid profile (total cholesterol, LDL, HDL, triglycerides)	The intervention group had a reduction in DII at 3 months, but no significant difference at 12 months. After 12 months, a 0.65 mg/L decrease in CRP and improvements in total cholesterol (−9.38 mg/dl) and LDL (−11.99 mg/dl) were observed among participants with greater DII reduction	Weight, height, BMI	Participants were overweight or obese; differences after the intervention were not highlighted	Health and lifestyle habits data collected via self-report and the perceived stress scale to measure stress levels	Stress and physical activity did not change significantly between groups. Observed changes in biomarkers were not mediated by these factors but rather by changes in DII
Derose et al. (24)	Not available	—	Body weight and BMI	A reduction in BMI and decreased weight gain were observed among participants in the intervention group compared to the control group	Not available	—
Miller et al. (26)	PHQ-4 (four items for depression and anxiety)	A slight reduction in distress was observed in the intervention group by week 9, but the effect was not sustained by week 15. The variation did not reach statistical significance	Self-reported weight, height, and BMI	The majority of participants were observed to be overweight or obese (65%)	Evaluated using the rapid version of the functional assessment of cancer therapy-general (FACT-G7)	Improvement in participants' quality of life after the interventions, with a positive impact on reducing fatigue and improving FACT-G7 scores
Diallo et al. (25)	Management of chronic diseases such as hypertension, diabetes, obesity, cardiovascular disease, and chronic obstructive pulmonary disease	Participants self-reported improvements in the management of chronic diseases	Not available	—	Self-report	Improvements in overall wellbeing and quality of life were reported, associated with increased intake of healthy foods and reduced social isolation
West et al. (27)	Not available	—	Not available	—	Not directly evaluated; explored qualitatively through interviews about wellbeing and self-esteem	Participants reported feeling more confident, proud, and motivated after the intervention. Many expressed improvements in psychological and social wellbeing
Begley et al. (28)	Not available	—	Not available	—	Not available	—
Reicks et al. (29)	Systolic and diastolic blood pressure	Significant improvement in systolic and diastolic blood pressure outcomes (*p* < 0.0001)	BMI and waist circumference	Significant weight loss and waist circumference reduction (*p* < 0.0001), with 54.1% of participants reducing their waist circumference	Qualitative self-report on wellbeing, satisfaction, overcoming obstacles, and motivation linked to food culture	Participants reported greater food awareness, enjoyment in cooking, and preparing meals with less salt and more vegetables. Reports indicated subjective improvements in wellbeing
Asher et al. (31)	Not available	—	Not available	—	Not available	—
Rainville et al. (32)	Not available	—	Not available	—	Not available	—
Williams et al. (34)	HbA1c levels, and self-care efficacy measures through SDSCA, DSMQ, and DTSQ	Improvement in diabetes management and quality of life among participants	Self-reported BMI and waist circumference	Reduction in BMI correlated with HbA1c reduction	Evaluated by the short form health survey (SF-12)	Improvement in the mental component score of quality of life
Ylitalo et al. (33)	Not available	—	Not available	—	Not specifically detailed, but related to the sense of community and belonging	Participants reported high levels of belonging and social support during the cooking classes
Begley et al. (30)	Not available	—	Not available	—	Not available	—
Barr-Porter et al. (36)	Mental health, measured using items from the CDC healthy days module (centers for disease control and prevention)	The study observed significant improvements in “days per month with poor mental health” and “days per month feeling worried, tense, or anxious”	Not available	—	Health-related quality of life (HRQOL) instrument was used to measure participants' quality of life	The study reported improvements in health-related quality of life, specifically in domains involving mental health such as sadness, depression, worry, tension, or anxiety
French et al. (37)	Not available	—	Not available	—	Not available	—
Kearsey et al. (35)	Not available	—	Not available	—	Food security measured by the household food security survey module (HFSSM)	Improvement in participants' level of food security was observed
Domper et al. (38)	Not available	—	Body weight, BMI, waist circumference, and hip circumference	The culinary intervention group (CIG) showed significant reductions in all anthropometric parameters compared to the nutritional intervention group (NIG)	Not available	—
Heredia et al. (39)	HbA1c levels, self-perception of health, and diabetes self-management skills; diastolic blood pressure	There was a significant reduction in HbA1c levels among participants, indicating improvements in diabetes control. Additionally, there were improvements in self-perception of health and diabetes self-management skills. No significant reduction in diastolic blood pressure was observed	Body weight and BMI	No significant changes in weight or BMI were reported	Self-report	Participants reported an improvement in quality of life, especially regarding mental wellbeing and self-perception of health

### 2.6 Outcomes

To characterize comprehensive food utilization, culinary workshops promoting the full use of fresh foods were considered, prioritizing healthier dietary habits. Identified strategies included culinary workshops that taught techniques for recipes using fresh foods.

These workshops also featured educational actions such as meal planning, safe and proper food storage guidelines, and practical recipes and techniques involving fresh and minimally processed foods.

## 3 Results

**Figure 1:**
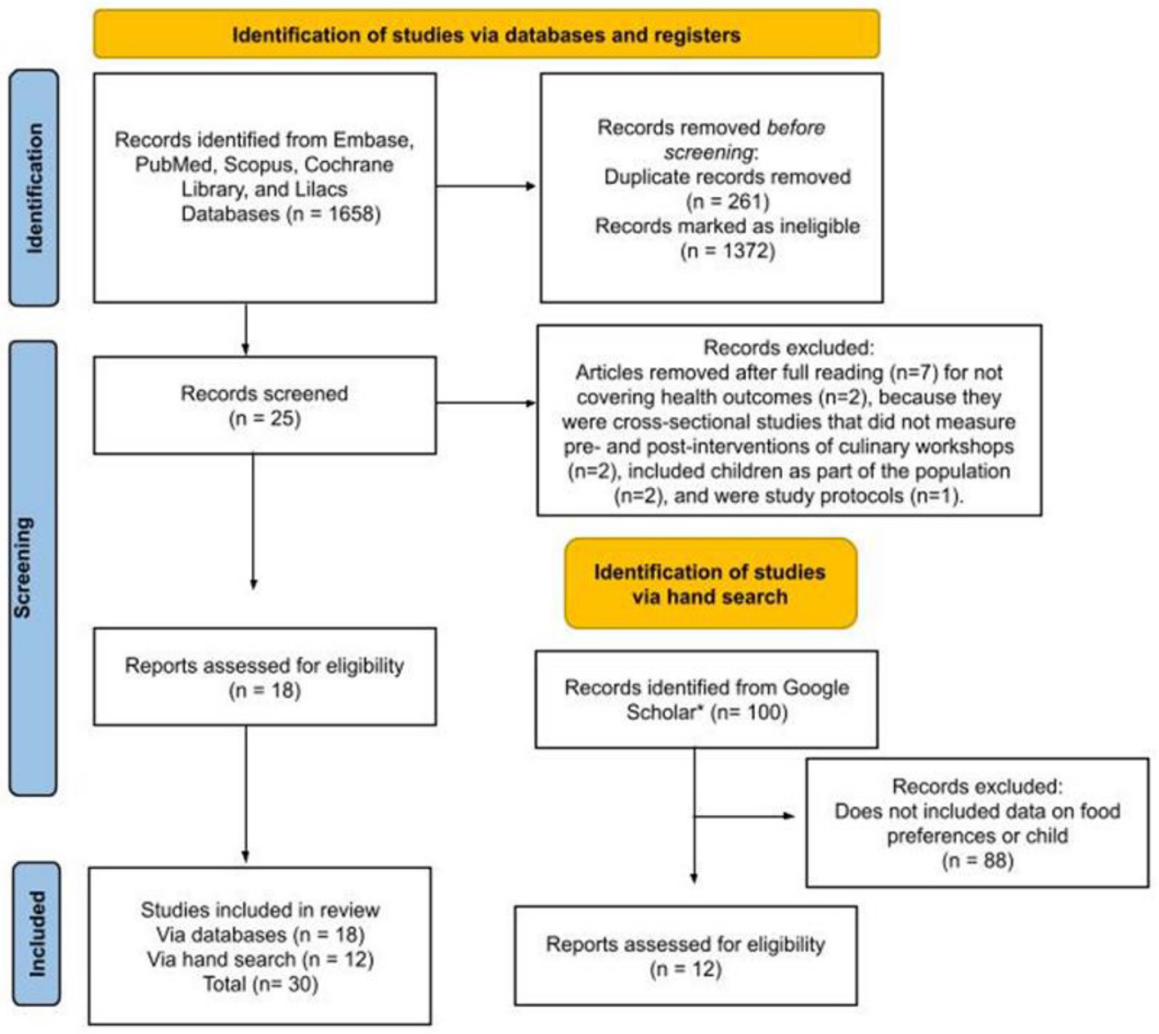
Flowchart of search and selection of studies for inclusion in the scoping review, based on the PRISMA-ScR.

The database search yielded 1,658 articles (Figure 1). After removing duplicate manuscripts (*n* = 261), 1,397 titles and abstracts were screened, resulting in the exclusion of 1,372 studies. An initial full-text analysis was conducted on 25 studies, of which seven were excluded for the following reasons: absence of health outcomes (*n* = 2), cross-sectional design without pre- and post- culinary workshop measurements (*n* = 2), inclusion of children as part of the population (*n* = 2), and being study protocols (*n* = 1). The Google Scholar search returned numerous results, sorted by relevance; only the first 100 entries were analyzed for inclusion. Following a careful review, 88 studies were excluded due to lack of data on dietary preferences or involving child populations. Ultimately, 30 studies were included for full-text analysis (11–40). The selected studies were published between 2002 and 2025, all of which were written in English.

Among the selected studies, the majority were conducted in the United States (60%) and Australia (16.66%), likely reflecting the research funding and infrastructure available in these countries. The United Kingdom (6.66%), Spain, Italy, Ireland, New Zealand, and Brazil each accounted for 3.33% of the studies (Table 1).

Analysis of the target groups revealed a strong focus on vulnerable populations, including individuals with low socioeconomic status, adults with preexisting health conditions (such as diabetes, cancer, and obesity), and ethnically diverse communities, notably Indigenous, African American, and Hispanic groups. Culinary and nutritional interventions were primarily implemented in community and healthcare settings, such as community centers, clinics, and food distribution organizations.

Table 1 summarizes the key characteristics of the studies included in this review, highlighting important details such as the location of the workshops, the populations involved, the delivery modes (whether in-person, virtual, or hybrid), and the frequency and duration of each intervention (Table 1).

Educational contexts, both formal (universities and hospitality schools) and informal (online courses), as well as farmers' markets and rural extension services, were also utilized, reflecting efforts to adapt interventions to local resources and emerging technologies. However, some studies lacked detailed descriptions of their intervention settings, highlighting a need for more contextual information in future research.

Regarding the format of the workshops, 80% were conducted in person, suggesting a preference for direct interaction, while 16.6% were delivered virtually and 3.3% used a hybrid format. This distribution reflects the growing adaptation to digital technologies, potentially accelerated by the COVID-19 pandemic. Sessions typically occurred weekly, lasting from 30 min to 3 h, with most programs spanning 4–8 weeks, although some extended up to 12 months or longer (Table 1).

In Table 2, we present the types of foods used in the workshops and the specific culinary preparations participants engaged with. Additionally, the table includes insights into food consumption patterns and participants' autonomy in making food choices (Table 2).

The analysis of ingredients used in interventions revealed a predominant focus on fruits and vegetables (83.3%), followed by whole grains (36.6%) and lean proteins (23.3%), with some studies referencing specific dietary patterns such as the Mediterranean and Atlantic diets. However, 16.6% of the studies did not provide detailed ingredient information, limiting the scope of analysis. The most common culinary preparations included salads (36.6%), soups (23.3%), and vegetable dishes (17.9%; Table 2).

Table 3 summarizes the health outcomes observed across the studies, focusing on changes in anthropometric measures, quality of life, and other clinical indicators. This overview is essential for understanding the broader impact of the culinary workshops, showing how they can contribute to improvements in both physical health and mental wellbeing (Table 3).

Among the studies included in this review, only fourteen reported clinical health outcomes. A significant reduction in glycated hemoglobin (HbA1c) levels and improved management of type 2 diabetes mellitus were observed following 6–12 weeks of culinary workshops (35, 40). A decrease in C-reactive protein (CRP) levels was also reported, associated with increased consumption of anti-inflammatory foods (24). Improvements in lipid profiles, particularly reductions in total cholesterol and LDL cholesterol, were identified in two studies (11, 16). Only one study directly measured blood pressure as a primary outcome, demonstrating statistically significant reductions in both systolic and diastolic blood pressure values (*p* < 0.0001) after a 6-week in-person intervention based on plant-based foods. Regarding mental health and wellbeing, several studies reported significant improvements, including reductions in the number of days experiencing feelings of sadness or anxiety (21, 26, 37) (Table 3).

Reductions in anthropometric parameters, such as body weight, BMI, and waist/hip circumference, were reported in 15 of the 33 studies. These outcomes were more commonly observed in interventions lasting 6 weeks or longer, with 53% showing statistically significant changes (11, 17, 25, 30, 35, 39) (Table 3).

Dietary autonomy was assessed in 81% of the studies, with robust and statistically significant evidence of improvements in confidence, culinary self-efficacy, dietary management, practical skills, and attitudes toward healthy eating (24, 27, 31, 34, 35, 39) (Table 3).

Regarding dietary intake, more than 90% of the studies demonstrated significant improvements in eating patterns, particularly with increased consumption of fruits, vegetables, whole grains, and minimally processed foods. Additionally, the interventions had a direct impact on reducing the consumption of added sugars, total calories, and ultra-processed foods (11, 15, 18, 20, 21, 23, 41) (Table 3).

Quality of life was assessed in seven studies, with statistically significant results observed in only three of them (11, 18, 26), which reported improvements in mental, functional, and emotional domains. The remaining studies provided qualitative evidence of enhanced wellbeing, health perception, and social engagement, particularly among vulnerable populations (Table 3).

## 4 Discussion

Culinary workshops have expanded their reach as an effective means to promote healthy eating habits and strengthen food and nutrition education (FNE). The results of this review directly corroborate this trend, highlighting that this type of intervention encompasses not only technical-cooking instruction but also a space for critical learning about food and its myriad possibilities, including the exchange of knowledge and cultural particularities that impact health, eating behavior, autonomy, and wellbeing.

In particular, although autonomy was not always directly measured, several studies documented improvements in culinary self-efficacy, confidence in food preparation, and greater independence in dietary choices. These findings support the interpretation of dietary autonomy as a multidimensional construct, often indirectly captured through behavioral and self-perception changes.

This analysis identified that eight studies reported statistically significant reductions in anthropometric parameters, such as body weight, BMI, and waist circumference. These findings reinforce the experiences reported by Freitas et al. (40), who used workshops as a training strategy for Nutrition students, evidencing that applied knowledge has the potential to positively influence participants' physical health. However, more robust clinical effects tend to emerge when workshops are combined with other systematic nutrition education actions and multidisciplinary approaches (41).

Although the presented data suggest the potential of workshops to positively influence metabolic health, the magnitude of the effects varies widely, as sustainability over the long term is often a determining factor (42). Therefore, there is a need to standardize protocols and assessments that address clinical and anthropometric parameters.

The results related to significant improvements in dietary intake are largely consistent with other studies found in the literature (7, 43), which describe culinary workshops as “sensitizing settings” capable of encouraging more conscious and sustainable eating practices. The study by Begley et al. (30), included in this review, showed significant gains in meal planning and preparation domains, reinforcing the idea that culinary practice is indeed a mechanism for food empowerment.

Moreover, as indicated by the data obtained from this synthesis and in line with the studies by Vaughan et al. (44) and Luz et al. (45), longer interventions tend to generate more consistent effects on eating behavior, as well as increased acceptance of new foods through repeated exposure and sensory engagement, favoring changes in food preferences.

Although data on quality of life were scarce among the included studies, the subjective effects reported on mental wellbeing, self-esteem, and positive health perception align harmoniously with findings related to the recovery of affective memories associated with cooking, the strengthening of social bonds, and the appreciation of culinary practices (46).

Among the aspects addressed in this review, it is confirmed that the duration of workshops is a fundamental factor for their effectiveness. Longer interventions, going beyond one-off meetings and offering follow-up or reinforcement of content, were associated with greater adherence, improvements in food consumption, and autonomy. Additionally, continuous approaches adapted to the realities of participants have proven to be much more effective, as they strengthen bonds and cultural appreciation (47). However, few studies conducted follow-up assessments, limiting the understanding of the long-term sustainability of the effects.

In Brazil, the promotion of healthy eating is supported by a robust set of public policies and structural programs. Among the most prominent are the National School Feeding Program (PNAE), which ensures balanced meals and nutrition education activities in public schools (48); the Dietary Guidelines for the Brazilian Population, an internationally recognized reference for its emphasis on fresh and minimally processed foods (49); and the Health in Schools Program (PSE), which integrates intersectoral actions focused on health and nutrition (50).

Additionally, local initiatives such as community gardens, solidarity kitchens, and university extension projects expand access to healthy foods and reinforce sustainable practices (51–53). These actions form an ecosystem of strategies aimed at improving food quality and promoting food and nutritional security, in which culinary workshops emerge as an educational and community tool with high potential for impact.

Despite growing interest in cooking workshops as a tool for improving eating habits and promoting health, there is still a significant gap in research focused on the impact of these interventions in developing countries such as Brazil. Most existing studies have mainly involved university students, a population that, although it shows beneficial results with these interventions, does not accurately reflect the broader demographic characteristics of most developing countries (22, 54). University students generally represent a more educated and higher-income group, which contrasts with the socioeconomic reality of the general population in countries such as Brazil, where lower levels of education, income disparity, and limited access to resources prevail (55).

While many successful interventions have been conducted in developed countries, the approach to culinary workshops can be adapted to the Brazilian context by considering local food habits, cultural diversity, and available resources. Workshops in Brazil, for example, could emphasize the use of traditional and locally available foods, making them more accessible and relevant to local populations. Additionally, adapting recipes to incorporate affordable ingredients and leveraging local knowledge on food preparation can increase the effectiveness and sustainability of these interventions (56).

Considering their multiple facets—from physical health to emotional wellbeing—culinary workshops should be integrated into public food and nutrition policies. Programs mentioned in the scope of the studies, such as “Cooking Matters” conducted in the United States, have already demonstrated applicability in vulnerable populations, while in Brazil, reported experiences (7, 43, 46) highlight the potential of workshops to strengthen fairer, more inclusive, and sustainable eating practices. It is essential to foster collaboration among universities, research centers, healthcare services, and social movements to support the implementation of culinary workshops as a health promotion tool, as they can become powerful pedagogical, community, and political instruments to tackle non-communicable chronic diseases and food insecurity.

Incorporating culinary workshops into public health initiatives in Brazil and similar countries requires a broader and more inclusive approach. Future studies should aim to include diverse populations, such as low-income communities, rural areas, and individuals with lower levels of formal education, to better capture the challenges and potential benefits for these groups. Additionally, adapting the content and delivery of culinary workshops to the specific needs and preferences of these populations, considering factors such as local food availability, cultural relevance, and economic constraints will be key to ensuring the success and sustainability of these interventions (57).

The standardization of culinary workshops can be achieved by establishing clear guidelines to ensure consistency across interventions. This includes defining specific objectives for each workshop, such as enhancing food autonomy or increasing the consumption of fresh and minimally processed foods. Moreover, it is essential to set recommended durations and frequencies for the workshops, with most programs running between 4 and 8 weeks to facilitate adherence and the practical application of learned concepts (14, 16, 18, 19, 24–26, 29, 31, 33–35, 38).

In addition, standardization should encompass the use of consistent assessment methods, such as self-report questionnaires and food diaries, which allow for comparability across different studies and contexts. Embracing digital platforms, increasingly prominent in the 21st century, is also a key strategy for expanding access to culinary workshops. Virtual and hybrid models offer flexible alternatives, particularly for individuals in remote areas or those with mobility challenges. The use of digital tools, such as apps and online videos, can further enhance the accessibility and adaptability of these interventions (58, 59).

Encouraging the participation of health professionals, such as nutritionists, during culinary workshops to provide accurate and personalized information is a way to ensure that participants receive appropriate and safe dietary guidance. Nutritionists can also play a key role in adapting recipes for specific health conditions, such as diabetes or hypertension, promoting healthy and safe eating. Integrating culinary workshops with other health services can enhance results and ensure continuity of care (60).

Another important aspect is the need to address food safety issues in culinary workshops, offering information about accessible and safe ingredients for consumption, as well as emphasizing food hygiene practices and allergy prevention (61, 62).

To incorporate culinary workshops into public policies in Brazil and similar countries, several mechanisms should be considered. First, integrating these workshops into existing programs such as PNAE or PSE could provide a framework for reaching large-scale populations. Workshops could be offered as part of nutrition education and health promotion initiatives, particularly in low-income and vulnerable communities, where access to nutritional education and healthy food is limited.

Another key mechanism would be aligning culinary workshops with local health and nutrition policies, ensuring they are incorporated into the broader strategy for improving diet quality and food security. Partnerships between universities, community organizations, and local governments could facilitate the implementation and scaling up of these workshops, while also considering the cultural, social, and economic realities of different regions.

## 5 Conclusions

Culinary workshops have shown positive impacts on health, but methodological limitations—such as short follow-ups and underrepresentation of studies in vulnerable settings—affect the generalizability of findings. Integrating these workshops into public policies through interdisciplinary, intersectoral, and community-based approaches can enhance their social relevance and long-term impact. Beyond cooking skills, workshops that embrace social and educational dimensions contribute to healthier, more equitable, and sustainable food practices.
