# Supplementary material for: From kitchen to health: how culinary workshops influence eating habits, autonomy, and wellbeing in adults–A scoping review
Source: Front Nutr. 2025 Aug 29;12:1653406. doi: 10.3389/fnut.2025.1653406 (PMC12425794; doi:10.3389/fnut.2025.1653406)
Supplement: Supplementary file 1 [file Table_1.docx]

Appendix A

Appendix A.1 – Search strategy

#1 "Cooking" [Mesh] OR "Cookery" OR "Cook*" OR "Culinary" OR "Culinary Workshops" OR "Cooking Classes"

#2 "Food Loss and Waste" [Mesh] OR "Food Waste and Loss" OR "Food Waste" OR "Waste, Food" OR "Food Loss" OR "Loss, Food" OR "Garbage" [Mesh] OR "Garbages"

Detail search:

("Cooking"[MeSH Terms] OR "Cookery"[All Fields] OR "cook*"[All Fields] OR "Culinary"[All Fields] OR "Culinary Workshops"[All Fields] OR "Cooking Classes"[All Fields]) AND ("Food Loss and Waste"[MeSH Terms] OR "Food Waste and Loss"[All Fields] OR "Food Waste"[All Fields] OR "waste food"[All Fields] OR "Food Loss"[All Fields] OR "loss food"[All Fields] OR "Garbage"[MeSH Terms] OR "Garbages"[All Fields] OR "Non-conventional food plants"[All Fields] OR "Unconventional food plants"[All Fields] OR "pancs"[All Fields] OR "Panc"[All Fields] OR "pancs"[All Fields] OR "Wild food plants"[All Fields] OR (("whole"[All Fields] OR "wholeness"[All Fields] OR "wholes"[All Fields]) AND ("statistics and numerical data"[MeSH Subheading] OR ("statistics"[All Fields] AND "numerical"[All Fields] AND "data"[All Fields]) OR "statistics and numerical data"[All Fields] OR "utilization"[All Fields] OR "utilisation"[All Fields] OR "utilisations"[All Fields] OR "utilise"[All Fields] OR "utilised"[All Fields] OR "utilises"[All Fields] OR "utilising"[All Fields] OR "utilities"[All Fields] OR "utility"[All Fields] OR "utilizations"[All Fields] OR "utilize"[All Fields] OR "utilized"[All Fields] OR "utilizer"[All Fields] OR "utilizers"[All Fields] OR "utilizes"[All Fields] OR "utilizing"[All Fields]) AND ("food"[MeSH Terms] OR "food"[All Fields] OR "foods"[All Fields] OR "food s"[All Fields])))

Search Strategy at EMBASE

#1 ‘cooking’/exp OR ‘cooking’/syn

#2 ‘kitchen’/exp OR ‘kitchen’/syn

#3 #1 OR #2

#4 ‘food waste‘/exp OR ‘food waste‘/syn

#5 ‘Garbage’

#6 #4 OR #5

#7 #3 AND #6

Search Strategy at Cochrane Library

#1 MeSH descriptor: [Cooking] explode all trees

#2 MeSH descriptor: [Food Loss and Waste] explode all trees

#3 MeSH descriptor: [Garbage] explode all trees

#5 #2 OR #3 OR #4

#6 #1 AND #5 0

Search Strategy at Virtual Health Library (LILACS)

#1 "Culinária" OR "Cooking" OR "Culinaria" OR "Cuisine (activité)"

#2 "Perda e Desperdício de Alimentos" OR "Food Loss and Waste" OR "Alimento Perdido y Desperdiciado" OR "Resíduos de Alimentos" OR "Garbage" OR "Residuos de Alimentos"

Detail search:

("Culinária" OR "Cooking" OR "Culinaria" OR "Cuisine (activité)" ) AND ("Perda e Desperdício de Alimentos" OR "Food Loss and Waste" OR "Alimento Perdido y Desperdiciado" OR "Resíduos de Alimentos" OR "Garbage" OR "Residuos de Alimentos" OR "Whole Utilization of Foods" OR "Aproveitamento Integral dos Alimentos" OR "Aprovechamiento Integral de los Alimentos" OR "Utilisation Totale de l'Alimentation")

Search Strategy at SciELO

("Culinária" OR "Cooking" OR "Culinaria" OR "Cuisine (activité)" ) AND ("Perda e Desperdício de Alimentos" OR "Food Loss and Waste" OR "Alimento Perdido y Desperdiciado" OR "Resíduos de Alimentos" OR "Garbage" OR "Residuos de Alimentos" OR "Whole Utilization of Foods" OR "Aproveitamento Integral dos Alimentos" OR "Aprovechamiento Integral de los Alimentos" OR "Utilisation Totale de l'Alimentation")
